# Characterizing US Spatial Connectivity and Implications for Geographical Disease Dynamics and Metapopulation Modeling: Longitudinal Observational Study

**DOI:** 10.2196/64914

**Published:** 2025-02-18

**Authors:** Giulia Pullano, Lucila Gisele Alvarez-Zuzek, Vittoria Colizza, Shweta Bansal

**Affiliations:** 1 Department of Biology, Georgetown University, 37th and O Streets NW, Washington, DC, 20057-1229, United States, 1 202 687 9256; 2 Fondazione Bruno Kessler, Trento, Italy; 3 Sorbonne Université, INSERM, Institut Pierre Louis d’Epidémiologie et de Santé Publique (IPLESP), Paris, France

**Keywords:** geographical disease dynamics, spatial connectivity, mobility data, metapopulation modeling, COVID-19, human mobility, infectious diseases, social distancing, epidemic, mobile apps, SafeGraph, SARS-CoV-2, coronavirus, pandemic, spatio-temporal, US, public health, mobile health, mHealth, digital health, health informatics

## Abstract

**Background:**

Human mobility is expected to be a critical factor in the geographic diffusion of infectious diseases, and this assumption led to the implementation of social distancing policies during the early fight against the COVID-19 emergency in the United States. Yet, because of substantial data gaps in the past, what still eludes our understanding are the following questions: (1) How does mobility contribute to the spread of infection within the United States at local, regional, and national scales? (2) How do seasonality and shifts in behavior affect mobility over time? (3) At what geographic level is mobility homogeneous across the United States?

**Objective:**

This study aimed to address the questions that are critical for developing accurate transmission models, predicting the spatial propagation of disease across scales, and understanding the optimal geographical and temporal scale for the implementation of control policies.

**Methods:**

We analyzed high-resolution mobility data from mobile app usage from SafeGraph Inc, mapping daily connectivity between the US counties to grasp spatial clustering and temporal stability. Integrating this into a spatially explicit transmission model, we replicated SARS-CoV-2’s first wave invasion, assessing mobility’s spatiotemporal impact on disease predictions.

**Results:**

Analysis from 2019 to 2021 showed that mobility patterns remained stable, except for a decline in April 2020 due to lockdowns, which reduced daily movements from 45 million to approximately 25 million nationwide. Despite this reduction, intercounty connectivity remained seasonally stable, largely unaffected during the early COVID-19 phase, with a median Spearman coefficient of 0.62 (SD 0.01) between daily connectivity and gravity networks.

We identified 104 geographic clusters of US counties with strong internal mobility connectivity and weaker links to counties outside these clusters. These clusters were stable over time, largely overlapping state boundaries (normalized mutual information=0.82) and demonstrating high temporal stability (normalized mutual information=0.95). Our findings suggest that intercounty connectivity is relatively static and homogeneous at the substate level. Furthermore, while county-level, daily mobility data best captures disease invasion, static mobility data aggregated to the cluster level also effectively models spatial diffusion.

**Conclusions:**

Our work demonstrates that intercounty mobility was negligibly affected outside the lockdown period in April 2020, explaining the broad spatial distribution of COVID-19 outbreaks in the United States during the early phase of the pandemic. Such geographically dispersed outbreaks place a significant strain on national public health resources and necessitate complex metapopulation modeling approaches for predicting disease dynamics and control design. We thus inform the design of such metapopulation models to balance high disease predictability with low data requirements.

## Introduction

Human mobility plays a crucial role in the spread of respiratory diseases [1]. The combination of regional travel and local commuting represents the spatial connectivity between locations, serving as the main driver in the geographic diffusion of infectious diseases. Characterizing the spatial dynamics of pathogen transmission is, therefore, intricately tied to unraveling human mobility patterns. Such a task has proven to be challenging due to the inherent complexity and privacy-related limitations on collecting mobility data [2]. Over the past few decades, researchers have extensively relied on mobility data obtained from census records, surveys, transportation statistics, commuting data, and international air traffic data. Such datasets have widely contributed to a better understanding of human mobility patterns and their impact on the epidemic spread [3-6], but can be limited in their resolution or scale. More recently, this gap has been filled by the use of mobile phone data [7,8], primarily based on phone records, but no such data have been available in the United States.

The global health crisis triggered by COVID-19 has underscored the critical need for swift access to mobility to help mitigate the spread of the virus. The urgency of the situation prompted an unprecedented sharing of data by private companies worldwide, through legally and ethically compliant agreements. These data were based on mobile location–based app usage and thus provided incomparable access to high-resolution, large-scale, and near-real-time mobility data that have expanded human mobility science [9] and computational epidemiology [10,11]. The availability of these data has especially represented a shift in the US public health and it has been used to inform epidemic models and reveal the impact of mitigation strategies on behavior [12-16]. Although the association between mobility patterns and COVID-19 transmission in the United States has been extensively explored, for example, in studies by Pei et al [14], Badr et al [16], Xiong et al [17], Tokey [18], Zheng et al [19], and Gao et al [20], no studies have been devoted to assessing when the underlying mobility network needs to be embedded into models to characterize epidemic spread.

Furthermore, the effects of control measures such as social distancing and travel restrictions as well as the most suitable geographical and temporal granularity for implementing these measures still lack clarity. This gap in understanding the characteristic spatio-temporal scale of mobility not only limits target control policies but also our ability to model transmission dynamics effectively. To date, mobility data have been integrated into epidemic models without due consideration for the optimal geographical (eg, municipalities, regions, and states) and temporal resolution (eg, day, week, and month) required to accurately capture epidemic spread. The level of granularity used in these models has consistently been dictated by a priori assessments from data providers [21,22].

To address these gaps, this study made 3 key contributions. First, for characterizing spatiotemporal scales, we systematically analyzed human mobility patterns across the United States using high-resolution mobile app–based location data to characterize intercounty connectivity at different spatial and temporal scales before and after the COVID-19 pandemic. Second, for quantifying mobility’s impact on the geographic spread of disease, we integrated these mobility data into spatially explicit transmission models to assess its role in the geographic diffusion of SARS-CoV-2 during the first wave. Finally, for evaluating the predictive power across scales, we analyzed how the predictive ability of epidemic models changes with varying resolutions of mobility data, identifying the spatial and temporal scales of intercounty connectivity that significantly influence disease dynamics. This analysis allowed us also to explore the trade-offs between using fine-grained mobility data and aggregated data at coarser scales.

While our approach yields valuable insights into human behavior and disease dynamics and makes a theoretical contribution to the field, we acknowledge its limitations—most notably the simplifications in the disease model, such as assuming homogeneous mixing within counties. However, our focus was on understanding heterogeneity at scales larger than counties, as most public health data are typically available at the county level. This approach represents a significant step toward understanding the role of mobility in geographical disease diffusion and optimizing the integration of mobility data into epidemic models to better inform public health policies.

## Methods

### Study Design and Population

This study investigated the role of human mobility at various temporal and spatial scales in the spread of COVID-19 across the US counties, using mobility and disease incidence data. First, we examined human displacements and the resulting intercounty connectivity patterns in the United States based on the daily number of visits between census blocks. We also analyzed disease incidence during the early phases of the COVID-19 pandemic, from January 2019 to July 2020, and used an inference framework to estimate underreporting based on the number of new deaths. In the second step, we developed a metapopulation model to simulate disease transmission, incorporating intercounty mobility patterns and epidemiological data, adjusted for underreporting. The study focused on US counties with populations greater than 10,000 to minimize biases in mobility and incidence data.

### Data Collection

#### Characterizing Intercounty Connectivity With Mobility Data

We used data from SafeGraph [23] (now Advan Patterns [Advan Inc]), a platform gathering mobility data from location-based mobile app usage. Specifically, we used the daily Social Distancing dataset provided by SafeGraph (refer to Multimedia Appendix 1 for dataset details). This dataset includes information on the number of mobile devices with a home from a census block group visiting another census block group or staying in the originating one for at least a minute. The data covered the period from January 2019 to April 2021 on a daily basis. These data were aggregated to the US county level to ensure consistency in geographic scale for disease surveillance and public health decision-making. A correction factor addresses spatial and temporal variations (Multimedia Appendix 1).

**Figure 1.**
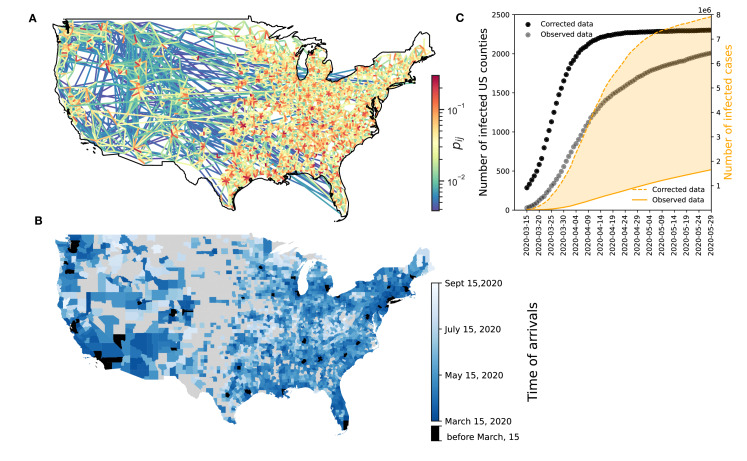
Data sources and epidemic context. (A) Mobility data. The figure shows the spatial connectivity network between US counties in March 2020. The map shows only links with the top 1% of coupling probability (p_ij_>.0038). (B-C) Public health data. (B) The map shows the time of arrival by county, defined as the time when the total number of cases (observed+unreported) in counties that reached at least 10 cases. Black-colored counties are counties that have been infected before March 15, 2020. (C) Black dots show the corrected number of infected counties over time nationally. Gray dots show the observed number of infected counties over time nationally. The orange solid line shows the daily number of new confirmed cases nationally, while the orange dotted line shows the real number of cases nationally accounting for underreporting.

We quantified monthly intercounty connectivity by normalizing visits from an origin county to destination counties and calculated average daily visits for all county pairs from January 2019 to March 2021. We then normalized the results so that the sum of probabilities for each county equals 1. The resulting time-evolving network reflected daily coupling probabilities. Figure 1A shows the connectivity network for March 2020. Figure S2 in Multimedia Appendix 1 compares monthly and daily datasets.

#### Characterizing Early Phase of COVID-19 in the United States With Public Health Data

The first confirmed case of COVID-19 in the United States emerged in Washington state on January 21, 2020, quickly leading to local transmission. Guidelines promoting social distancing and discouraging gatherings were issued on March 16, 2020. While many European countries enacted nationwide restrictions, the United States implemented localized measures at varying times. Lockdowns peaked in the United States in April 2020, with over 40 states enforcing stay-at-home or shelter in-place orders [24]. Despite these efforts, COVID-19 continued to spread, with most US counties reporting cases by June 2020. As cases surged again in October 2020, recommendations for social distancing were made to keep epidemic activity in check.

The COVID-19 disease incidence data were derived from the Centers for Disease Control and Prevention (CDC) data [24]. We used daily reports of new cases and the timing of their arrival in each US county, defined as the day when at least 10 cases were recorded in that area. Daily new-reported cases and time of arrivals were adjusted for potential underreporting at the county level, estimated using global data on COVID-19 cases and fatalities, as outlined in a study by Russell et al [25] (Figures 1B and 1C).

### Statistical Analysis

#### Describing Temporal and Spatial Variability in the Mobility Network

We examined the monthly network structure to evaluate the temporal mobility patterns, quantifying each county’s degree (number of connections to other counties). We also defined link persistence as the probability links with nonzero mobility in a month of 2019 that remained in the same month of 2020 and 2021. We also fit a gravity model to the intercounty connectivity network for each month. Community detection analysis using the stochastic InfoMap algorithm developed by Rosvall et al identifies regions with more frequent internal movements [26]. We used a bootstrap resampling method to account for stochasticity (refer to Multimedia Appendix 1 for details). Urban and rural classifications are based on the National Center for Health Statistics Urban-Rural Classification Scheme. All network analyses were performed using Python’s NetworkX library [27], which is widely used for the creation, manipulation, and study of the structure, dynamics, and functions of complex networks. Gravity model fitting was conducted using the scikit-mobility library, a Python package designed for mobility data analysis [28].

#### Incorporating Human Mobility Into Infectious Disease Models

We used a stochastic non-Markovian transmission model with a metapopulation structure at the US county level [21]. The model accounts for disease transmission proportional to (1) infected residents not moving, (2) infected visitors coming from other counties, and (3) returning residents previously infected in other counties. The resulting force of infection in the county *i* is defined as follows:


$$\lambda_{i}=\lambda_{ii}+\sum_{i\neq j}\lambda_{ji}^{v}+\sum_{i\neq j}\lambda_{ij}^{r}$$

$$\lambda_{ii}=\beta p_{ii}^{2}\frac{I_{i}}{\hat{N_{i}}}$$

$$\lambda_{ji}^{v}=\beta \;p_{ii}p_{ji}\frac{I_{j}}{{\hat{N}}_{i}};\;\lambda_{ij}^{r}=\beta \;p_{ij}\frac{{\hat{I}}_{j}}{{\hat{N}}_{j}}$$

where *p_ij_* is the coupling probability extracted from the intercounty connectivity network. The effective population, and effective number of infections are, respectively, defined as follows:


$$\hat{N_{i}}=p_{ii}N_{i}+\sum_{i\neq j}p_{ji}N_{j}$$

$$\hat{I_{i}}=p_{ii}I_{i}+\sum_{i\neq j}p_{ji}I_{j}$$

We considered the susceptible-exposed-infectious-recovered epidemic dynamics specific to COVID-19. The epidemics parameters were described by Pullano et al [29].

The detailed mathematical framework, model calibration, and implementation details can be found in Multimedia Appendix 1. The model and inference framework were developed in C++ to optimize performance and efficiency, particularly when working with large-scale data.

#### Inference Framework and Goodness of Fit

To calibrate the epidemic pathway, we used the cumulative number of infected US counties (Figure 1C). Calibration covered March 14 to July 15, 2020, when all counties reported infections. Parameter estimates *β_pre_*_−*LD*_ (March 15‐31) and *β_post_*_−*LD*_ (March 31-May 15) were derived using maximum likelihood, assuming a Poisson distribution for reported cases.

We assessed model performance by comparing the modeled invasion probability *p_i,inv_*(*t*) with the observed early phase COVID-19 spatial invasion. *p_i,inv_*(*t*) denotes the probability for a county *i* reporting at least 10 infected cases on day *t* in the simulation. The goodness of fit is defined as follows:


$$G(t)=\sum_{i}\left(I_{i}\log p_{i,\text{inv}}(t)+\left(1-I_{i}\right)\log\left(1-p_{i,\text{inv}}(t)\right)\right)$$

*I_i_*=1 if the county *i* reported at least 10 infected cases at the day *t*; *I_i_*=0 otherwise.

#### Comparing Models Across Geographical Scales

In designing the metapopulation structure at different spatial scales, we aimed to better understand the role of mobility heterogeneity that matters in disease diffusion. Beyond this primary goal, this approach also highlighted the data requirements necessary for accurate modeling at different spatial scales. Specifically, it explored the implications of using mobility data at a coarser regional scale (eg, cluster or state), assuming higher-resolution flow data are unavailable.

For a given spatial scale *R* (eg, cluster and state), we randomized the mobility links among counties within a region *R*, preserving the number of links. Coupling probabilities for connected counties within each region *R* are equal to the average coupling probability of the links within that region, as follows:


$$p_{ij}^{R}=\frac{\sum_{l,k\in R}p_{lk}}{N}$$

with *i,j,* ∈, and *R*, while coupling probabilities across regions are not changed.

### Ethical Considerations

This study used publicly available, deidentified, and aggregated data from SafeGraph (now Advan Patterns) and the CDC. SafeGraph ensures user privacy by implementing differential privacy techniques, which involve adding Laplacian noise to anonymize data at the Census Block level. In addition, SafeGraph excluded connections associated with census block groups containing data from fewer than 2 devices. Ethical review for this study was sought from the Institutional Review Board at Georgetown University and the study was approved on October 14, 2020 (STUDY00003041).

## Results

Analyzing the US county connectivity via mobility data revealed temporal and geographical stability, identifying the key scale driving COVID-19’s initial spread. Our findings addressed public health needs and determined the optimal scale for metapopulation model design.

### Temporal Stability of the Intercounty Connectivity Network

**Figure 2.**
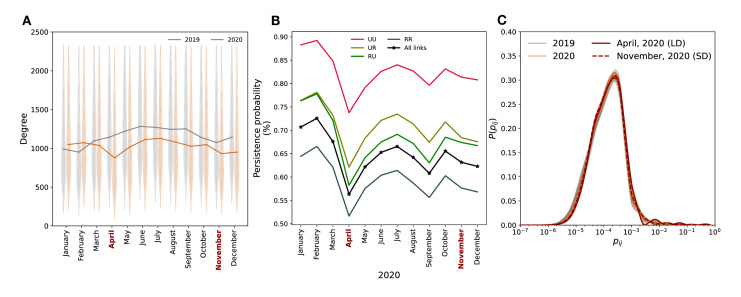
Temporal stability in intercounty mobility. (A) The monthly degree distribution of the intercounty network is shown as a violin plot with 95% CIs. It demonstrates significant variability while remaining consistent across months. (B) The persistence probability of links is illustrated, denoting the likelihood that a connection existing in 2019 remains present in 2020 and 2021. The plot provides a breakdown for different link types: urban-urban (UU), urban-rural (UR), rural-urban (RU), and rural-rural (RR) links. (C) Distribution of coupling probabilities in the connectivity network by month. We highlighted in dark red: April, 2020 (LD) that represents the peak time of number of US states in lockdown, and November, 2020 (SD) that represents the period when social distancing recommendations were in place. LD: lockdown; RR: rural-rural; RU: rural-urban; SD: social distancing; UR: urban-rural; UU: urban-urban.

From January 2019 to March 2021, mobility remained relatively stable, except for a significant drop in April 2020 coinciding with lockdown measures, reducing daily visits from 45 million to around 25 million nationwide (Figure 2A). The mobility shock extended throughout the month, encompassing a transitional period (Figures 2A and 2B). Analysis of the intercounty connectivity network’s temporal evolution revealed a consistent seasonal pattern in degree distribution and link persistence. Only April 2020 showed local variations, with a 23% reduction in degree (from 1144 to 877) compared with 2019 and a 20% reduction in link persistence (from 0.70 to 0.56) compared to previous months in 2020. Surprisingly, no variation occurred in November 2020, despite strong social distancing recommendations preceding the winter surge of SARS-CoV-2. The reduction in rural-urban connections was particularly pronounced, with a 22% decrease (from 0.75 to 0.58). The decrease stabilized in May and beyond. Notably, urban-urban connections exhibited greater resilience over time when compared with connections involving rural areas. We hypothesized that rural-rural connections were systematically less persistent due to the inherently less stable nature of these links. Factors such as lower population density, reduced economic activity, and less frequent interconnectivity in rural areas contributed to this instability. Furthermore, while coupling probabilities stayed consistent over the study period (Figure 1C), the probability of staying in the home location exhibited larger variability (Multimedia Appendix 1).

Despite occasional local fluctuations, the intercounty connectivity network demonstrated temporal stability and high predictability through a gravity fit model (Figures S7-S9 in Multimedia Appendix 1). The Spearman coefficient between the original and modeled intercounty connectivity network remained constant over time, with a median of 0.62 (SD 0.02).

### Spatial Stability of the Intercounty Connectivity Network

**Figure 3.**
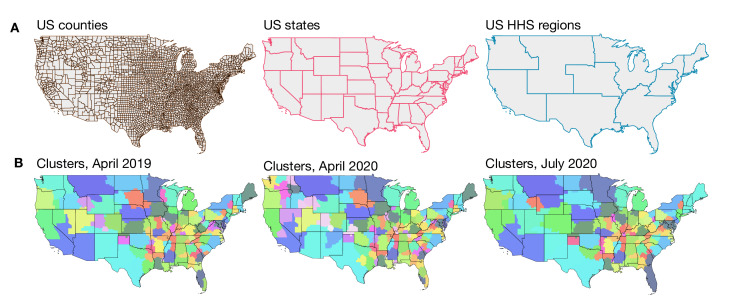
Analysis of spatial stability. (A) Geographical subdivisions at the county and state levels within the United States, as well as the division into Health and Human Services regions, used for health administration purposes. (B) We partition the intercounty mobility network so that each cluster of counties is more strongly connected to each other via mobility than to counties in other clusters. We find highly consistent portioning based on mobility networks from April 2019, April 2020 (during the mandated lockdown period of the early COVID-19 pandemic), and July 2020 (after the lockdown period of the early COVID-19 pandemic). Clusters are colored to delineate cluster boundaries and do not represent any other information. Counties colored in gray have populations of fewer than 11,000 inhabitants and are excluded from the analysis. HHS: Health and Human Services.

To identify the geographic scale at which mobility is highly connected, we detected clusters of counties that were more connected via mobility within the cluster than outside the clusters, we used a network community detection algorithm. Our hypothesis was that this partitioning of the United States would be at a geographic scale larger than 3143 US counties but smaller than 50 US states or 10 Health and Human Services (HHS) regions. Indeed, we found that based on human mobility, the United States can be partitioned into around 100 regions that split most US states into multiple clusters (Figure 3). We also found that these clusters were highly and spatially contiguous and respected state boundaries (with a similarity measured by normalized mutual information as 0.82). Furthermore, these regions demonstrated stability over time (normalized mutual information=0.95) despite the perturbations of the early phases of the COVID-19 pandemic (Figure 3B and in Figures S9-S10 in Multimedia Appendix 1). Thus, we identified a persistent geographic partitioning of the United States in which clusters were more connected within than between, and hypothesized that the relevance of mobility to the spatial diffusion of infectious diseases occured at a mesoscale.

### Implications for Metapopulation Disease Models

After examining the stability of mobility patterns over time and space, we evaluated how spatiotemporal scales of mobility affect disease modeling in a metapopulation framework by integrating connectivity networks into a model simulating the initial SARS-CoV-2 spread in the United States. We investigated the influence of geographic scale by homogenizing networks at different spatial levels, maintaining the county resolution. In addition, to gauge the impact of temporal scale, we provided the model with either a time-evolving or static connectivity network representing mobility patterns since March 2020, considering the network’s temporal stability. Goodness of fit was assessed by comparing predicted and observed disease arrival times in counties.

**Figure 4.**
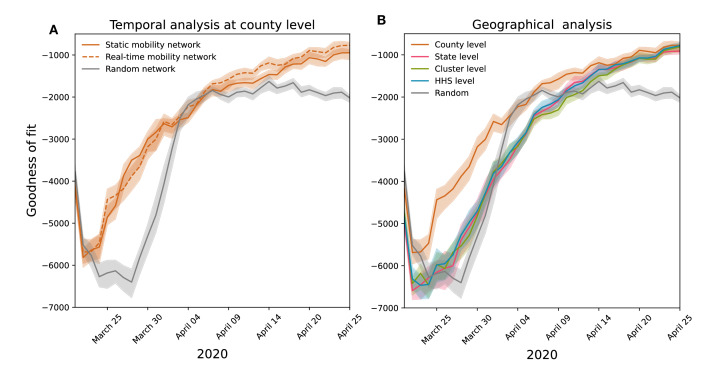
Implications of temporal and spatial scale of mobility data for the prediction with metapopulation disease models. (A) The goodness of fit (median and 95% CI) for the time of arrivals for metapopulation models at a county level, informed by a time-evolving intercounty connectivity network, a static intercounty connectivity network, and a random intercounty network. (B) Solid lines show the goodness of the fit (median and 95% CI) for metapopulation models informed with state-, cluster-, and Health and Human Services–level static mobility network. HHS: Health and Human Services.

Figure 4A shows that a county-level metapopulation model using empirical connectivity networks accurately predicts early COVID-19 diffusion, outperforming a Erdős–Rényi random network. Indeed, the empirical mobility network had a stronger goodness of fit throughout the early phase of the pandemic. This emphasized the crucial role of human mobility in the spatial spread of the initial SARS-CoV-2 wave and underscored the importance of mobility data for modeling SARS-CoV-2 spread. In addition, predictions based on static and time-varying mobility networks were comparable, suggesting that static data were sufficient to accurately capture epidemic spatial heterogeneity. Following the initial invasion phase in March, transmission became more widespread, reducing the importance of spatial connections. At this stage, random networks and county-level networks yielded similar performance, indicating that local epidemic growth outweighed the influence of importations and exportations. Figure 4B shows that county-level mobility data predicted spatial diffusion better than data at larger scales, such as US HHS regions, states, and clusters (as defined in Figure 3). All larger-scale data performed similarly to a random network, indicating they lack the granularity needed to capture diverse mobility patterns effectively. Sensitivity analyses on the definition of the time of arrivals and its impact on the goodness of fit are reported in Multimedia Appendix 1, more precisely in Figure S14B.

## Discussion

### Principal Results

Our findings revealed significant insights into the dynamics of human mobility and their implications for infectious disease modeling. In contrast to findings from other countries (eg, France [29], India [30], Germany [31], and Spain [32]), we observed that despite the implementation of local social distancing measures and lockdowns, intercounty connectivity remained largely unperturbed, leading to rapid geographic diffusion of SARS-CoV-2. Mobility patterns experienced only marginal changes before and after the early-stage COVID-19 pandemic. The most notable disruption occurred during the first lockdown period in April 2020, when mobility sharply declined. Although a temporary reduction in mobility was observed, it proved to be short-lived as mobility patterns quickly returned to prerestriction levels. Importantly, this decline in mobility did not alter the underlying intercounty connectivity structure, potentially diminishing the overall effectiveness of the implemented travel restrictions. Even when mobility is limited—resulting in low-strength edges between locations—these connections can still facilitate the spread of disease, introducing pathogens into new areas [33]. This underscores the challenge of relying solely on travel restrictions as a mitigation strategy, as they may fail to disrupt the pathways that enable disease transmission, particularly when the connectivity network remains intact. Such insights emphasize the need for more nuanced approaches that address the persistence of network structures during public health interventions. Notably, even during periods of social distancing recommendations, the mobility network remains relatively stable. Assuming the lockdown represents the most extreme form of mobility disruption, the temporal stability findings suggest that global human mobility demonstrates resilience against short-term changes.

We also assessed the spatial stability of the intercounty connectivity network by detecting spatial communities based on mobility patterns. Our results indicate that mobility-driven clusters align closely with state boundaries, reflecting the influence of administrative and geographical factors on human movement, accordingly with Steinegger et al [31]. These clusters exhibited remarkable stability over time, reinforcing the idea that spatial mobility patterns are deeply ingrained and relatively resistant to abrupt changes. The fact that mobility patterns are highly correlated with state boundaries suggests that state-level structures could be effective for designing target public health interventions based on travel reductions. Our findings underscore the importance of considering mobility patterns when designing interventions, resource allocation, and disease control strategies.

As shown in the context of COVID-19 pandemic in France [34], we also demonstrated that incorporating high-resolution human mobility data are crucial for accurately capturing the spatial spread of infectious diseases. Our findings indicate that county-level, daily mobility data offer the most accurate representation of the spatial spread of disease in the United States. Notably, static county-level mobility data achieve similar model performance to real-time data, suggesting that an undisturbed representation of reality is adequate for reproducing spatial spread. More interestingly, our exploration of various spatial scales for metapopulation models underscores the significance of aligning the model’s structure with the inherent spatial scale of human mobility. While county-level mobility data yield the most accurate depiction, mobility databased clusters, US states, and HHS regions do not capture the heterogeneity of the COVID-19 geographical diffusion.

### Limitations

While our study provides valuable insights, it is not without limitations. Our work focused on the early phase of the pandemic, during which response measures (eg, social distancing, closures, and lack of masking) were largely homogeneous in the United States, and pharmaceutical measures (eg, vaccination and antivirals) were not available; thus, these findings are not generalizable to later stages. Furthermore, we assume homogeneity within the US counties. In addition, Safegraph mobility data, like all mobile app–based location data, exhibit sampling biases. Ongoing efforts to comprehend these biases are crucial for developing better correction methods [35]. An independent analysis by Safegraph revealed the underrepresentation of older and non-White individuals in point of interest-specific analyses, though the panel is representative of race, education, and income [35,36].

### Comparison With Previous Work

Since the onset of the COVID-19 pandemic, mobile phone data have played a crucial role in addressing the public health crisis [10,11,13-15,22,29,30,34,37]. During this period, numerous network operators and private enterprises have made considerable efforts to swiftly share their data within the confines of legal regulations. Consequently, researchers worldwide have embarked on working with this data, monitoring human behavior caused by containment measures and adaptive responses to the epidemic, and using it to enhance epidemic models in order to increase their reliability [14,15,22,29,34].

While static mobility data have predominantly been analyzed and integrated into models over the past decades [3,4], the current accessibility to real-time human behavior data prompts an essential investigation into the optimal scenarios for using this dynamic information versus relying solely on static representations of reality [38]. Equally important is the exploration of the characteristic mobility scale to comprehensively capture the intricate coupling between different locations, a consideration with potential implications for target control policies to reduce epidemic activity, and for improving epidemic model forecasting. Furthermore, numerous researchers have emphasized the pressing necessity to implement standardized strategies that facilitate rapid data access while upholding stringent data privacy measures [2]. To address this gap in the literature, in this study, we investigated the spatial connectivity of the US counties during the early phase of the COVID-19 pandemic using high-resolution real-time human mobility data obtained from mobile phone usage.

### Conclusions

While characterizing the key role of mobility in the spatial invasion of the COVID-19 pandemic in the United States, our study sheds light on the global stability of human mobility patterns, and the relevant information needed to design a reliable predictive model. This result may be specific to countries, such as the United States, in which mobility restrictions were not stringent, specified for intercounty mobility, nor enforced. Metapopulation models that incorporate accurate mobility data can provide valuable insights into disease dynamics and enhance our ability to predict and control the spread of future infectious disease outbreaks. Furthermore, standardized data extraction and sharing that we introduced might help facilitate the timelines associated with legal agreements for data sharing, which do not always align with the rapid spread of epidemics, thus diminishing the feasibility of timely responses to such outbreaks.

## Supplementary material

10.2196/64914 Multimedia Appendix 1 Supplementary material.

## Abbreviations

CDC: Centers for Disease Control and Prevention
HHS: Health and Human Services
