# Supplementary material for: Characterizing US Spatial Connectivity and Implications for Geographical Disease Dynamics and Metapopulation Modeling: Longitudinal Observational Study
Source: JMIR Public Health Surveill. 2025 Feb 18;11:e64914. doi: 10.2196/64914 (PMC11856803; doi:10.2196/64914)
Supplement: Multimedia Appendix 1 [file publichealth-v11-e64914-s001.docx]

# Data source

## Mobile App-based location data


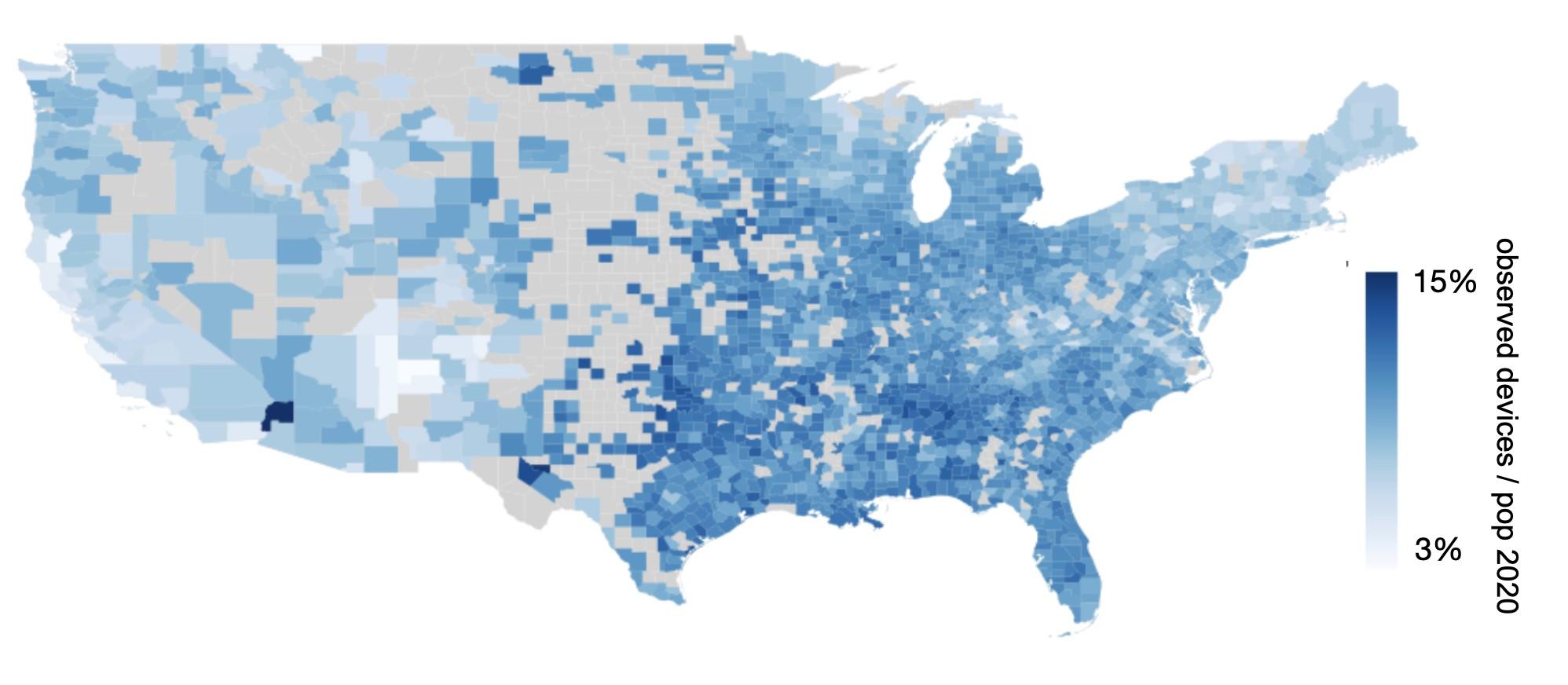


**Figure S1.** Mobile app-based location data coverage. Color-coded map of the ratio between observed mobile phone devices and population in 2020 by US county.

To address the spatial and temporal heterogeneity in the observed devices obs*_i_* within each county *i* (Figure S1), we developed a correction factor:


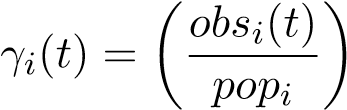


where *pop_i_* is the population in the county *i*. To reduce sampling biases, we exclude the bottom 25% of counties by population size, running all analyses on 2327 geographical counties within the continental US of a population size greater than 11,000.


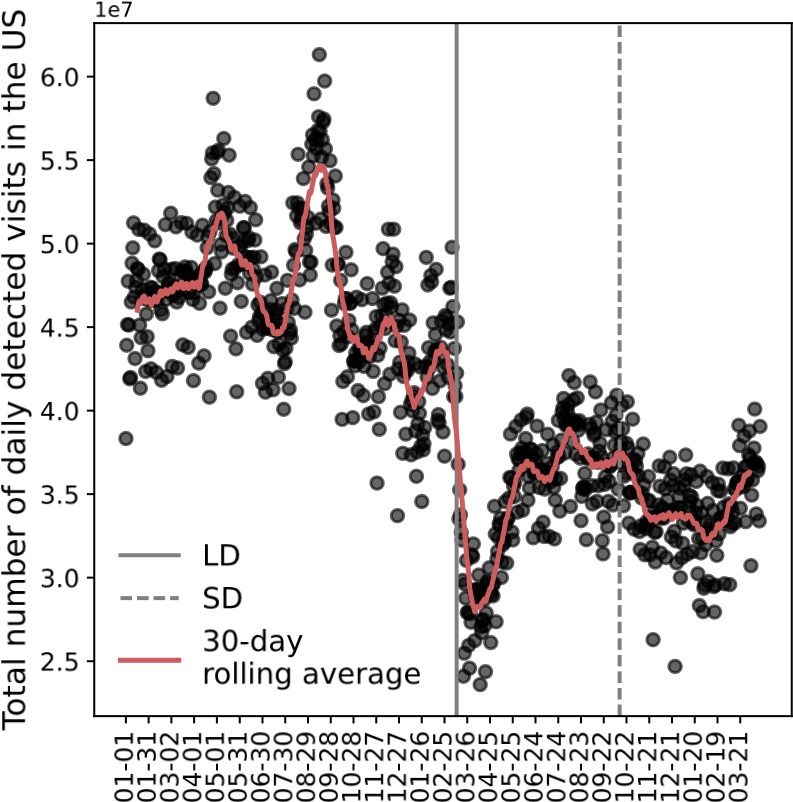


**Figure S2.** Detected mobility over time. Black dots show the daily total number of detected visits in the US provided by social distancing dataset. Solid brown lines show the 30-day rolling average. Vertical gray lines show Lockdowns (LD), and social distancing timelines (SD), respectively. Total number of devices revealed in any location summarized at the national level. The daily average is 40935499, with a 25% reduction at the end of March 2020 due to COVID-19 intervention policies.

## Comparing Neighbors patterns and social distancing dataset

SafeGraph’s Neighborhood Patterns [1] and Social Distancing [2] dataset both contain footfall data aggregated by census block group (CBG) in the U.S. While the Social Distancing dataset does not account for any filtering procedure, Neighborhood Patterns (NP) does not report data unless at least 2 visitors are observed from census block groups. 48% of connections in SD are not present in the NP dataset. As the Figure shows, the filtering process cut long-range connections, which are important from an epidemiological perspective. For this reason, we decided to use the daily SD dataset.

# Additional results on the temporal stability

**Characterization of intercounty connectivity network**


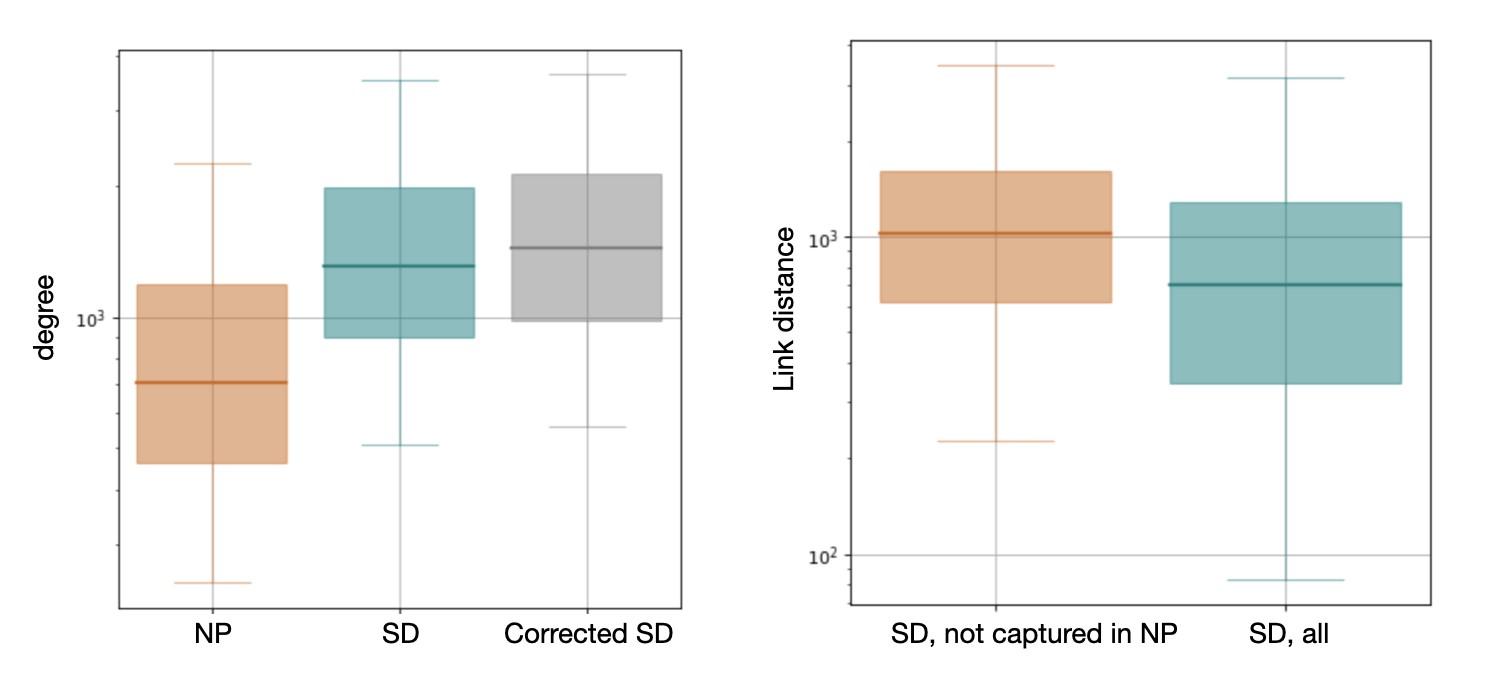


**Figure S3.** Comparison between SafeGraph’s Social Distancing (SD) and Neighborhood Patterns (NP) dataset. Left plot: Box plots indicate the 95% reference range of the degree distribution in the intercounty connectivity network extracted using the NP dataset, the SD dataset, and the SD dataset corrected for heterogeneity in data coverage. Box plots indicate the 95% reference range of geographical distances between county connections in the SD network. The plot is broken down, accounting for all links and only the links not captured by the SD dataset.


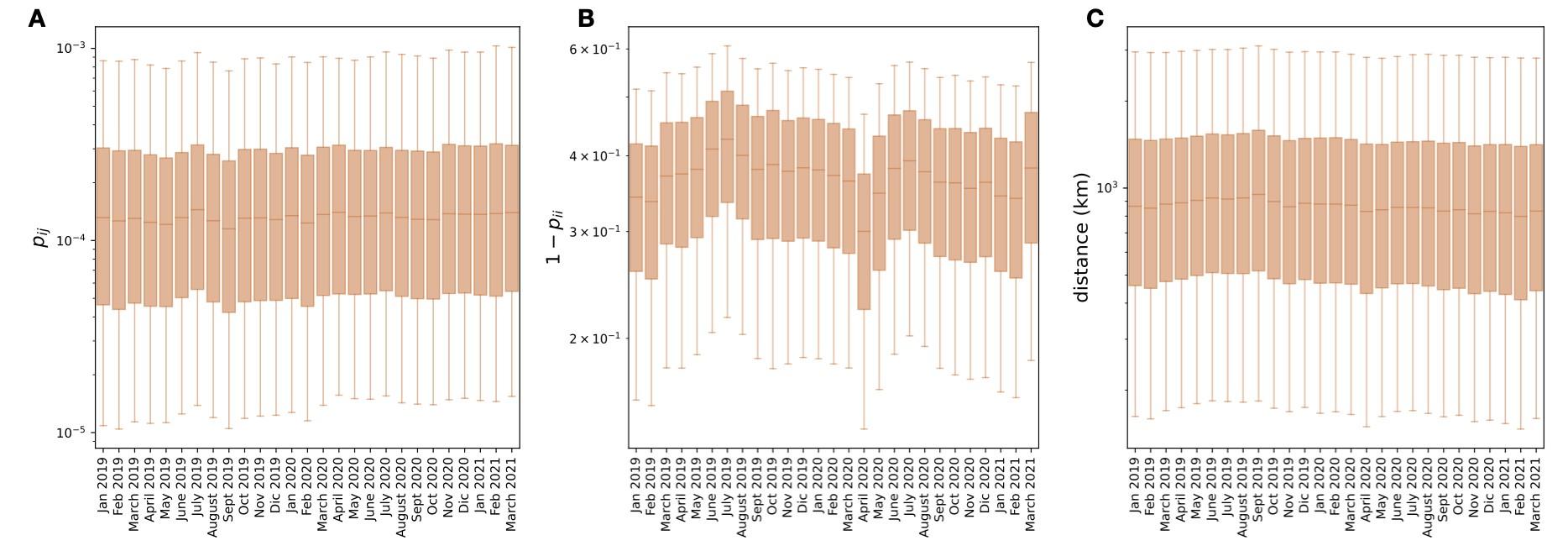


**Figure S4.** Temporal Stability of intercounty connectivity network. (A) The monthly connectivity between any pair of counties *i*, *j*, called coupling probability *p_ij_*. Boxplots account for the 95th percentile of the distributions. (B) The monthly probability of going out of the residential counties (self-loop in the inter-county connectivity network). (C) Geographical distance between connected counties in the network. Boxplots account for the 95th percentile of the distributions.


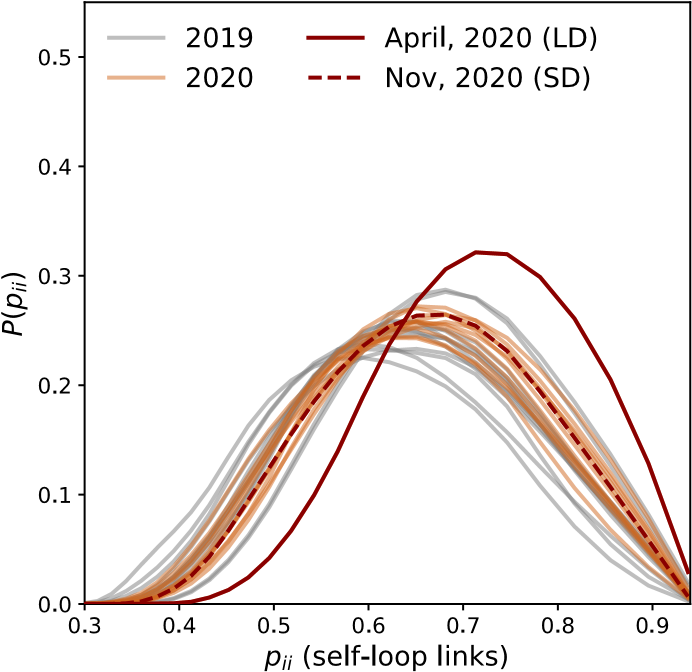


**Figure S5.** Probability of staying in the county of residence. The monthly probability of being in the residential counties (self-loop in the inter-county connectivity network). Theprobability of not moving during lockdowns in April, 2020 increased as expected.

## Gravity Model

The gravity model is defined as follows:


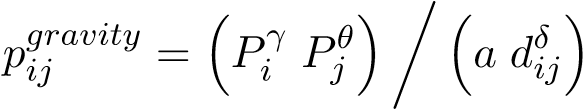


where *p_ij_* is the coupling between two US counties *i* and *j*, and *γ*, *θ*, *δ* are the estimated parameters through the intercounty connectivity network. *P_i_* and *P_j_* are the population of the counties *i* and *j*, respectively.

**A B**


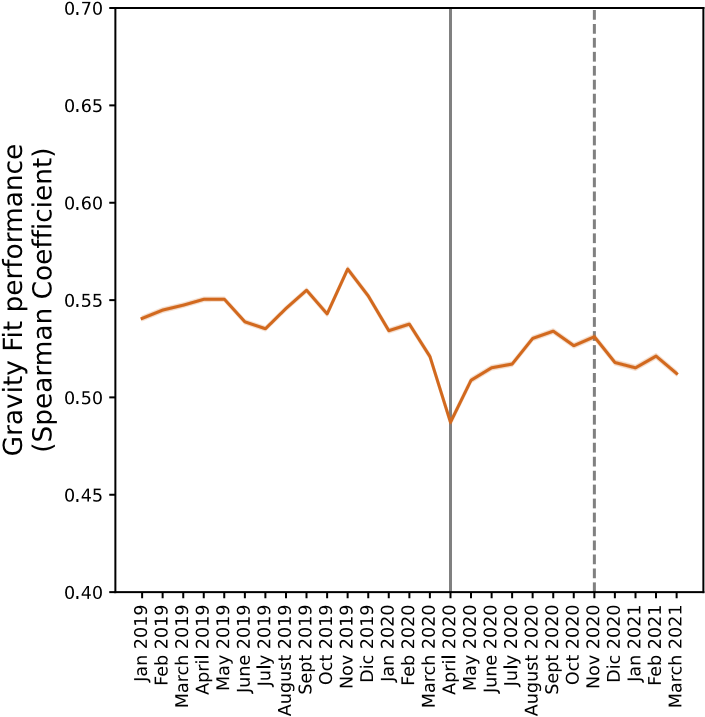

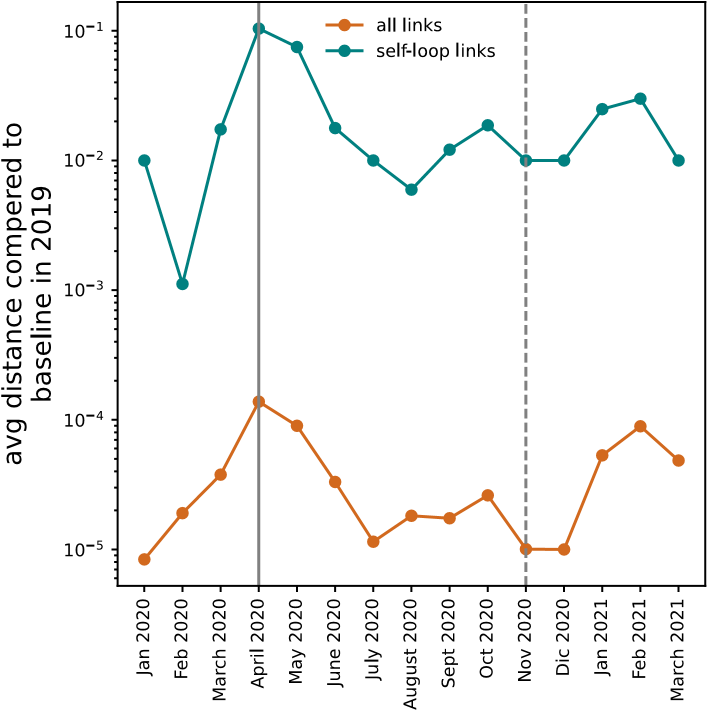


**Figure S6.** Connectivity network analysis over time. A) Average distance between the connectivity network links in 2020, 2021 with the same month in a pre-pandemic period in 2019. B) Spearman Coefficient of the monthly Gravity Model. The Spearman coefficient over time illustrates the correlation between the observed intercounty connectivity network and the gravity network fitted using the observed intercounty connectivity network.


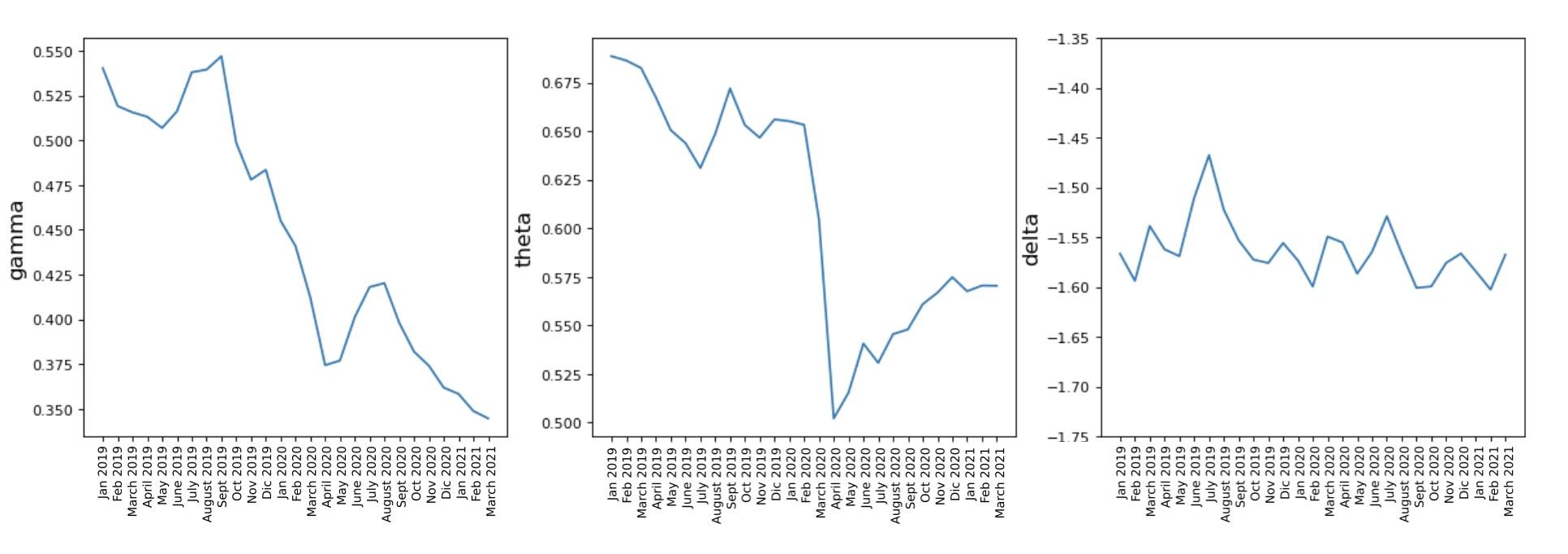


**Figure S7.** Fitted parameters of the monthly Gravity Model.

#
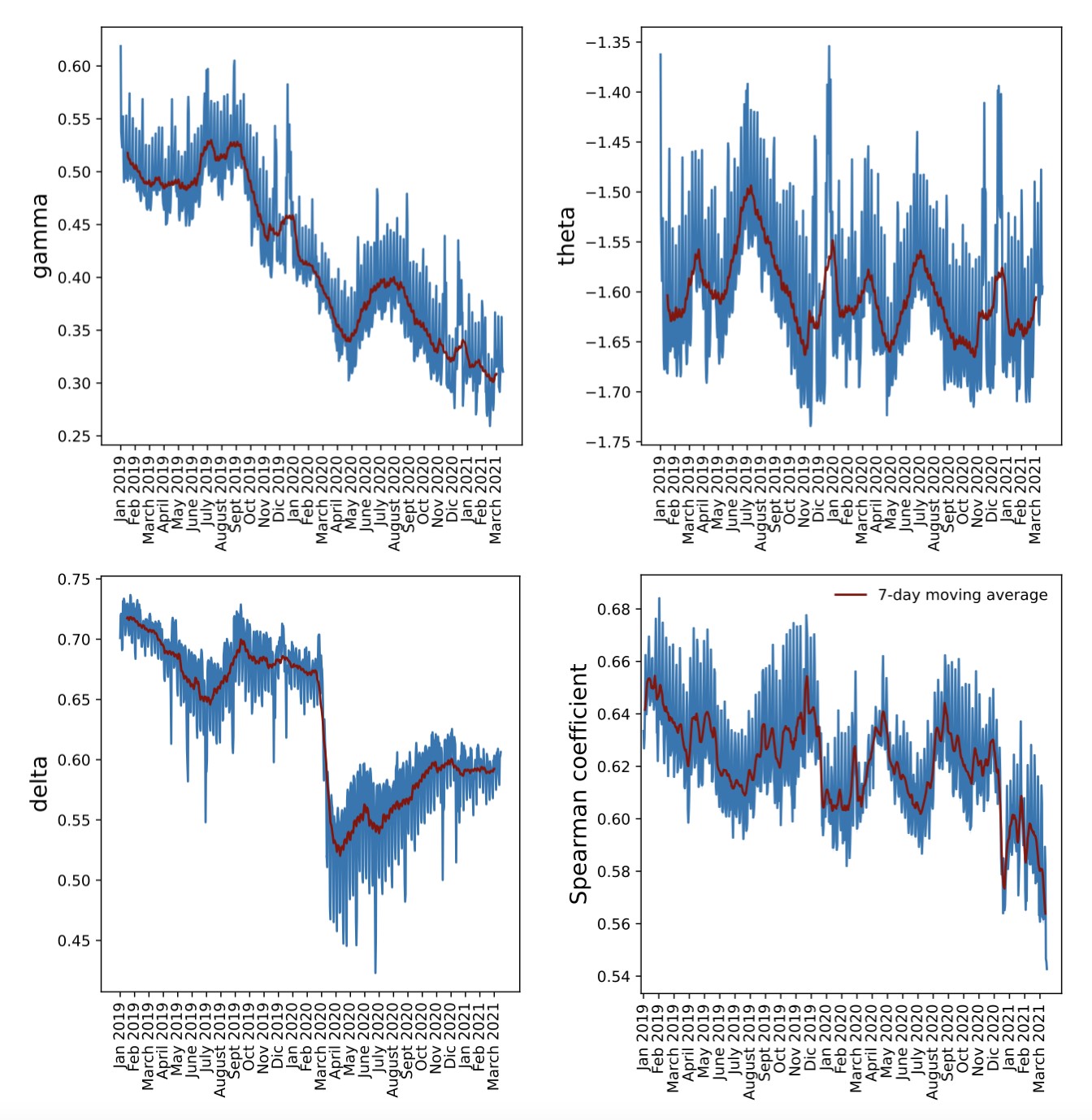


**Figure S8**. Fitted parameters and Spearman Coefficient of the daily Gravity Model. The Spearman coefficient over time illustrates the correlation between the observed intercounty connectivity network and the gravity network fitted using the observed intercounty connectivity network.


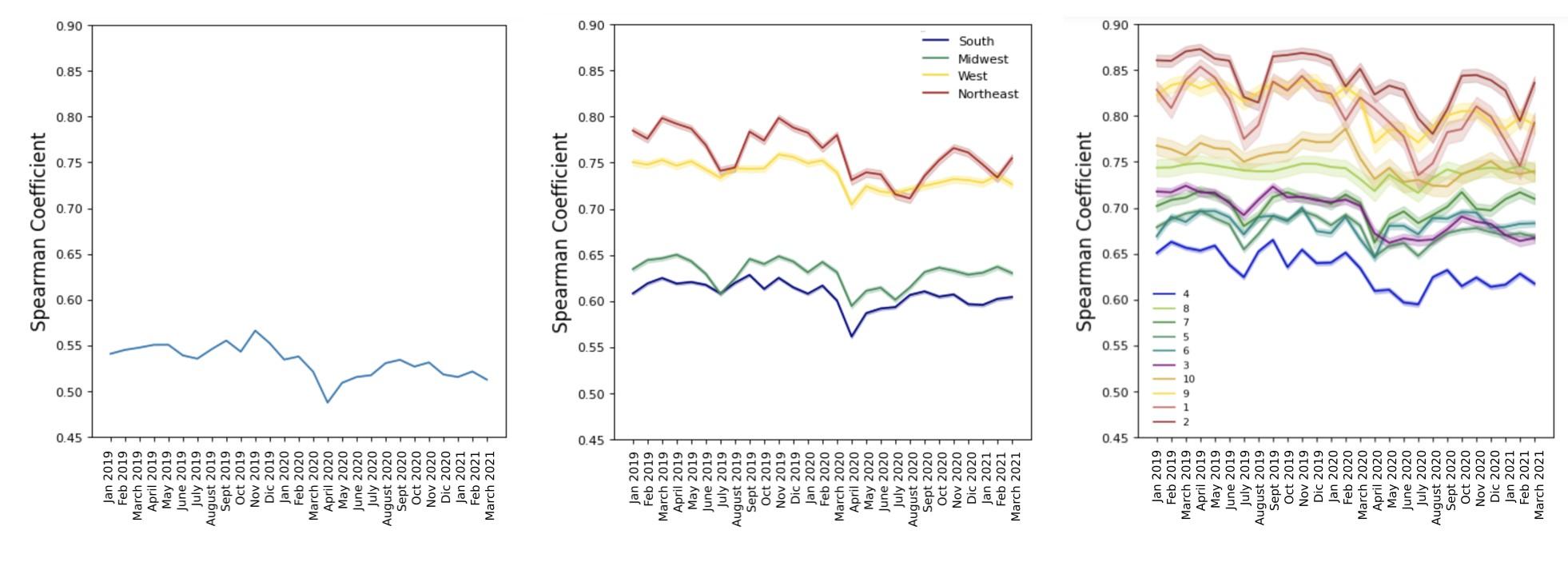


**Figure S9.** Spearman coefficient over time. The plots depict the performance of the fit across different spatial resolutions, including national, regional, and HHS office scales, respectively.

# Additional results on the spatial stability

To characterize spatial stability, we used INFOMAP algorithm. INFOMAP was developed particularly for mobility fluxes, and it uses the map equation and an information-theoretic approach, assuming that observed mobility flows are governed by a random walk process.


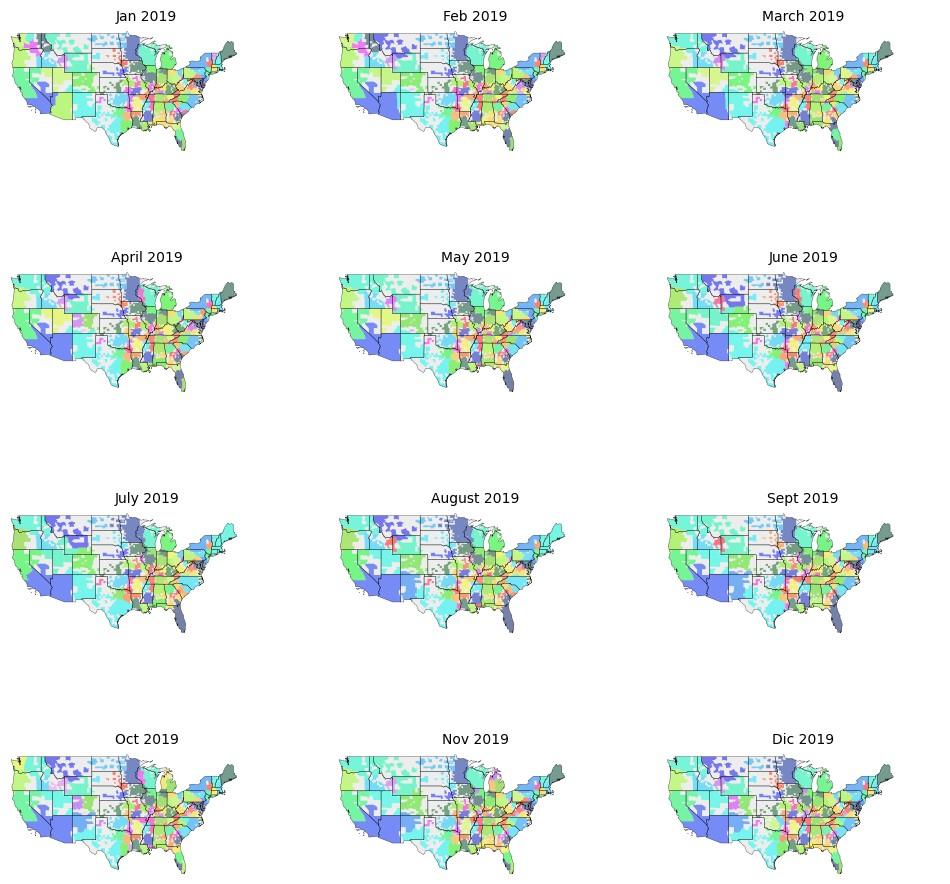


**Figure S10**. Monthly Infomap clusters in 2019.

## Clustering analysis accounting for INFOMAP stochasticity

In order to assess the accuracy of the INFOMAP community detection algorithm, we compute 25 simulations starting from the same seed. Then we compute the best partition of the system. Besides, we compare the performance of INFOMAP with the Louvain clustering algorithm in Figure S12 finding a better characterization for the temporal evolution of the clusters with INFOMAP. In the case of Louvain, we sample 25 copies of the original network *G*, and for each copy, we draw for each edge a new weight, which is given from a random Poisson distribution with mean in the original weight. Then, we compute the best partition of the system.


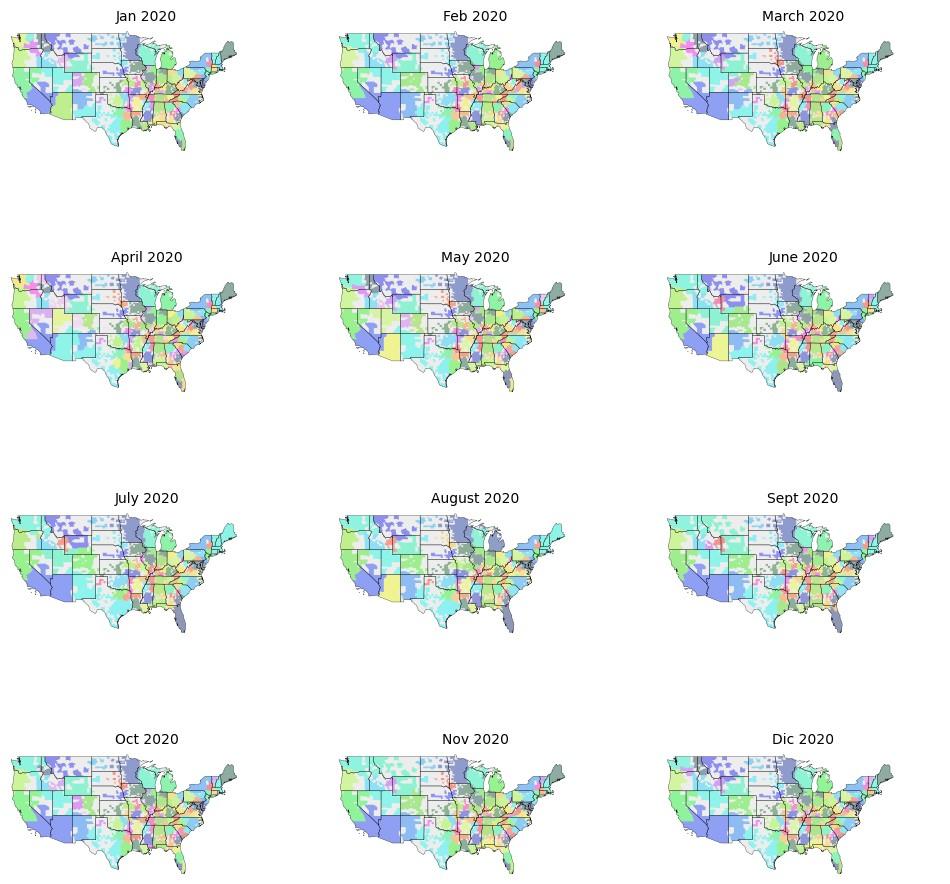


**Figure S11**. Monthly Infomap clusters in 2020.


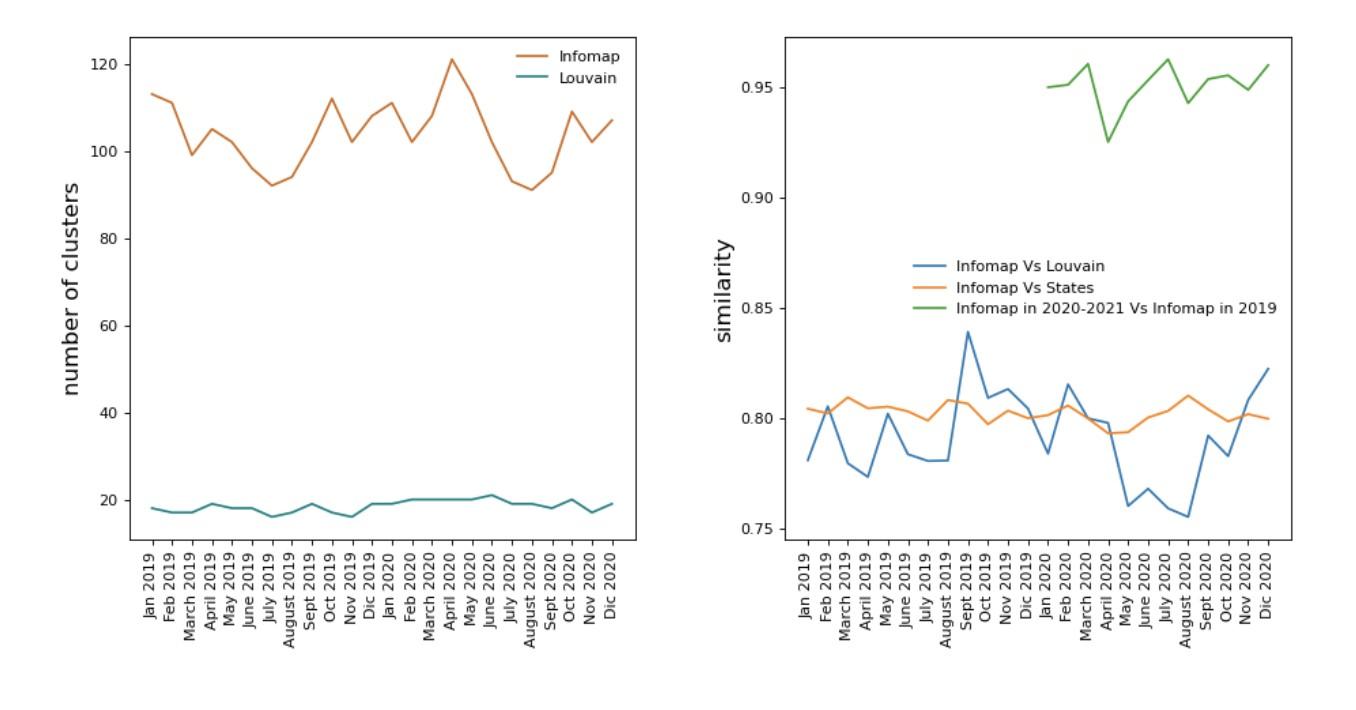


**Figure S12.** Cluster analysis. Left plot: number of clusters detected by Infomap and Louvain clustering algorithms. Right plot: monthly similarity between cluster repartitions.

## Simulation details and Inference framework

The initiation of stochastic simulations is set for March 15, 2020. This date is deliberately chosen to focus exclusively on the national-scale invasion, disregarding any preceding international multi-seeding events as outlined in [3]. The model was initialized on reported cases on March 15, 2020, with necessary adjustments made to account for underreporting. Counties with reported cases before March, 15 are shown in Figure 1B in the main manuscript. Underreporting was computed as defined in [4], and is shown in Figure 1C in the main manuscript. For each simulation, the model outputs are the time of arrival of the first 10 infected cases in any county and the daily number of counties reached by the epidemic. A total of 60 stochastic simulations are performed using identical initial conditions. For the purpose of analyzing simulation outcomes, we compute the invasion probability *p_i,inv_*(*t*) for every county *i* and time *t*. This probability denotes the likelihood of the epidemic reaching county *i* by time *t* [5]. It is calculated by dividing the number of runs in which county *i* is impacted by the epidemic by the total number of runs conducted. In order to quantify the 95% confidence intervals (CIs) for the invasion probability, 60 runs were sampled with replacement 100 times, and relevant statistics were computed accordingly. The model is calibrate from March 14 to July 15, 2020, when all counties reported infections. We estimate the parameters *β_pre_*_−_*_LD_* (March 15-31) and *β_post_*_−_*_LD_* (March 31-May 15) using a maximum likelihood defined as follow:

$$L\left( Nc_{data} | \beta_{\left\{ pre-LD \right\}},\beta_{\left\{ post-LD \right\}} \right)=\prod_{t} \left( Nc_{data}(t) | Nc_{model}\left( t \right) \right)$$

Where $Nc_{data}(t)$ are the number of counties at the time $t$ reporting at least 10 infected cases corrected by the underreporting, and $Nc_{model}\left( t \right)$ is the predicted number of counties at the time $t$ reporting at least 10 infected cases.

## Sensitivity analysis

## Baseline

## To validate the choice of March as the baseline, we also tested February as an alternative. The goodness of fit remains stable across these different baselines.

##

**Figure S13**. Baseline Sensitivity Analysis.
The goodness of fit (median and 95% confidence interval) for the time of arrival predictions from metapopulation models at the county level is shown with March as the baseline (brown curve) and February as the baseline (teal curve).

## Time of arrivals

We recalculated the goodness of fit by varying the case count threshold used to define the timing of arrivals. As illustrated in Fig. S14A, when the threshold is very low (e.g., th=5), the random model achieves better performance because the arrival times are too noisy to be accurately characterized. With a higher threshold (e.g., th=20), we delay the time of arrivals, which consequently delays the time window during which the county-level model outperforms other methods and the goodness of fit is not informative anymore for our focus on the early-stage invasion. Conversely, Fig. S14B demonstrates that analyzing the rank order of arrivals yields consistent results regardless of the selected threshold and support our results showing that county-level outperform the goodness of fit, while all the other resolution perform as the random network. This is because ranks are independent of time, and the relative order of arrival times remains preserved across different thresholds.

**Figure S14.** Sensitivity Analysis for the definition of time of arrivals. The plots illustrate the goodness of fit (median and 95% confidence interval) for time-of-arrival predictions generated by metapopulation models, evaluated under different thresholds for defining arrival times (t=5, 10, 15 infected cases). Figure S14A presents the goodness of fit calculated using the actual arrival times, while Figure S14B shows the goodness of fit based on the rank order of arrival times.

# References

1. *Neighborhood Patterns — SafeGraph. Link: https://docs.safegraph.com/docs/neighborhoodpatterns*.
2. *Social Distancing Metrics — SafeGraph. Link: https://docs.safegraph.com/docs/social-distancingmetrics*.
3. Sen Pei, Sasikiran Kandula, and Jeffrey Shaman. “Differential effects of intervention timing on COVID-19 spread in the United States”. In: *Science Advances* 6.49 (2020), eabd6370. ISSN: 2375-2548. DOI: 10.1126/sciadv.abd6370.
4. Timothy W. Russell et al. “Reconstructing the early global dynamics of under-ascertained COVID-19 cases and infections”. In: *BMC Medicine* 18.1 (Oct. 2020), p. 332. ISSN: 1741-7015. DOI: 10.1186/s12916-020-01790-9.
5. Michele Tizzoni et al. “On the Use of Human Mobility Proxies for Modeling Epidemics”. In: *PLOS Computational Biology* 10.7 (July 2014), e1003716. . ISSN: 1553-7358. DOI: 10.1371/journal.pcbi.1003716.
